# The Role of the Phylogenetic Diversity Measure, PD, in Bio-informatics: Getting the Definition Right

**Published:** 2007-02-19

**Authors:** Daniel P. Faith

**Affiliations:** The Australian Museum, 6 College St., Sydney, NSW, Australia, 2010

**Keywords:** PD, phylogenetic, diversity, conservation, evolutionary history, feature diversity, biodiversity

## Abstract

A recent paper in this journal (Faith and Baker, 2006) described bio-informatics challenges in the application of the PD (phylogenetic diversity) measure of Faith (1992a), and highlighted the use of the root of the phylogenetic tree, as implied by the original definition of PD. A response paper (Crozier et al. 2006) stated that 1) the (Faith, 1992a) PD definition did not include the use of the root of the tree, and 2) Moritz and Faith (1998) changed the PD definition to include the root. Both characterizations are here refuted. Examples from Faith (1992a,Faith 1992b) document the link from the definition to the use of the root of the overall tree, and a survey of papers over the past 15 years by Faith and colleagues demonstrate that the stated PD definition has remained the same as that in the original 1992 study. PD’s estimation of biodiversity at the level of “feature diversity” is seen to have provided the original rationale for the measure’s consideration of the root of the phylogenetic tree.

## Introduction

Recent papers in this journal (Crozier et al. 2005; Faith and Baker, 2006) have discussed some of the emerging bioinformatics issues associated with the applications of PD (phylogenetic diversity) calculations for biodiversity assessment. The highlighted PD applications have ranged from those applied to phylogeny of closely related species (Faith 1992a; Faith and Baker, 2006) to those using current taxonomy as a proxy for hard-to-get phylogenetic patterns (following Faith, 1992a,Faith, 1992b; Faith, 1994a,Faith, 1994b).

A new paper in this journal (Crozier et al. 2006) now has raised additional questions about the definition of PD, and has made two false claims:

1. that the Faith (1992a) original definition of PD *did not* imply the use of the distance to “the root of the complete tree,” and
2. that Moritz and Faith (1998) *changed* the definition of PD, following work by Nee and May (1997), to include the distance to the root.

Here, I show that both of these claims are false.

## I. The Faith (1992a) Definition of PD Did Take the Root into Account

### The Faith (1992a) example of PD comparisons

**Figure 1:**
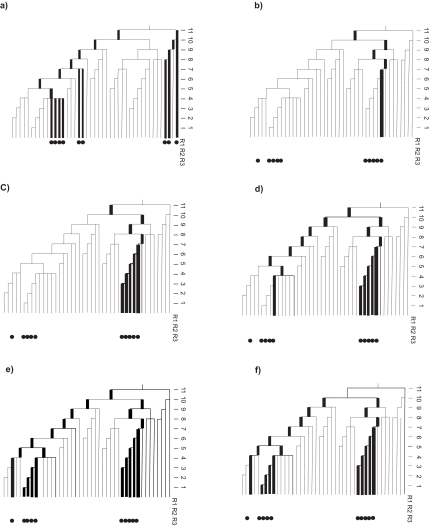
An expanded worked example from Faith (1992a), based on phylogenetic tree for bumble bees (*sibiricus* group within *Bombus*), that shows how the Faith (1992a) definition and application of PD used the root of the tree. The example compared 3 different sets of taxa, tabulating the PD for each (Table 2 in Faith 1992a). In each case, bold branches indicate those counted by PD for the particular calculation. Numbers at the right of the tree indicate the scale for branch lengths. a) the PD evaluation of set R1. b) initial branch length addition for the R3 cluster on the right side of the tree. c) complete calculation of the PD tally for the right-most cluster. d) initial branch length addition for the clusters on the left of the tree. e) complete calculation of the PD tally for the left-most clusters. f) an incorrect evaluation of set R3, in which the common root of the complete tree is ignored.

Faith (1992a) defined the PD (“phylogenetic diversity”) of a set of selected/protected taxa as the sum of all branches on the phylogenetic tree that spans that set. Here, I will re-examine the very first real-world example of PD, presented in that 1992 study (reproduced here as Fig. 1). This worked example from Faith (1992a) will clearly show how the Faith (1992a) definition and application of PD used the root of the tree. Further, this expanded presentation of the calculations from Faith (1992a) hopefully will provide a useful summary of the PD rationale and definition for future occasions when a worked PD example is needed.

Figure 1 displays the phylogenetic tree for bumble bees (*sibiricus* group within *Bombus*) reproduced from Faith (1992a). The example compared 3 different sets of taxa, tabulating the PD for each (Table 2 in Faith, 1992a). I will begin with the calculations for the first set, referred to as “R1” in Faith (1992a). We will count up the total PD by summing up branch lengths, starting from the right hand side. This is easily done using the branch length scale at the right of the tree, as in Faith (1992a). The first taxon (first dot in the diagram) yields a count equal to the full 11 units (Fig. 1a). Then, the count for the addition of the second dot discounts branches already used, and so yields an additional 10 units. The next dot (taxon) yields 8 more units. Next, in moving to the left side of the tree, the first dot from that cluster contributes a count of 11 additional units. Once again, further additions discount branches already tabulated, with the subsequent dots contributing 7, 7, 4, 4, and 4 units. The total PD of R1 is 66 units, corresponding to the value for the set R1 shown in Faith (1992a; Table 2).

These calculations included the base or root of the tree. Of course, we might have used some even more distant ancestral root for the tree, and obtained a larger PD index value of, say, 100+66 or 10,000+66. A PD calculation for R1, considered on its own, only requires *some* root ancestral to all those taxa (forming, for purposes of the analysis, the “complete tree”). In providing an index value, naturally there is no need to count back to the root of “all life.” Following the calculation of the PD value for the set R1, analyses might have proceeded in the usual way to calculate the losses in PD corresponding to various scenarios of species losses from the set (Faith, 1992a,Faith, 1992b; Faith et al. 2004).

At the same time, any PD *comparison* of alternative sets requires some common root appropriate to the full range of calculations–the root of the “complete” tree implied by all those taxa. Faith (1992a) illustrated exactly this point in the comparison of the PD value for set R1 and set R3.

Figure 1b–e shows the detailed calculation of PD for alternative set R3. Again, proceeding from right to left, the first dot (taxon) provides a count of 11 units (Fig. 1b). The next 4 taxa in that cluster yield 6, 5, 4, and then 3 additional units (Fig. 1c). Next, we move on to the cluster of 4 taxa to the left on the tree. The first dot yields 10 more units (Fig. 1d). The next dot yields only 3 units, because we do not double count already represented branches. Similarly, the next two dots yield 2 units and 1 unit. Finally, the left-most taxon provides an additional 5 units of branch length (Fig. 1e). The total PD of set R3 is 50 units, corresponding to the reported value for set R3 in Faith (1992a; Table 2).

Note that, if the PD calculations in this context had ignored “the root of the complete tree” defined by these comparisons, then the calculation for R3 would be that depicted in Figure 1f. Such a calculation would have wrongly represented the PD of set R3 as not capturing the deeper branch at the top of the tree, resulting in an *incorrect* value of 49 units (*not* the correct value of 50 units reported in Faith (1992a, Table 2)). Thus, the original PD example in Faith (1992a) illustrates how the root of the complete tree is properly taken into account under the corresponding PD definition provided in that paper.

### The Faith (1992b) examples

**Figure 2:**
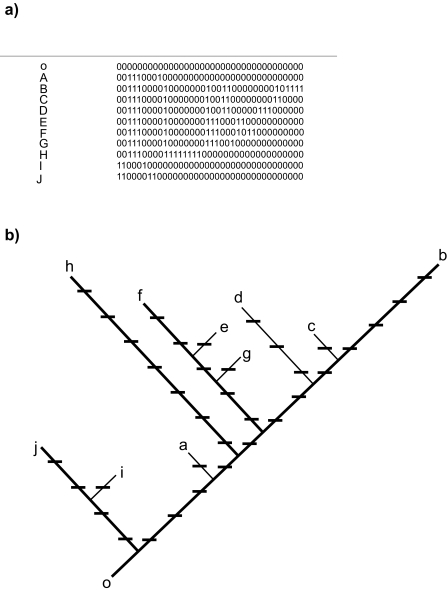
An example reproduced from Faith (1992b), that highlighted the clear the links from the PD definition to estimation of “feature diversity” and the inclusion of the root of the tree. a) The hypothetical data from Faith (1992a,Faith 1992b), in which rows are taxa and columns record features. Given the all-0 outgroup, 1-states indicate new features. b) The hypothetical tree or cladogram from Faith (1992a,Faith 1992b) for taxa a through j and outgroup O. The inferred derivations of new features from the data matrix are recorded by tick marks along branches. Given these branch lengths, PD calculations then reflect numbers of features for different sets of taxa.

The re-visited example above makes it clear that the Faith (1992a) definition of PD did take the root of the “complete” tree into account. Further, this use of the root was not an arbitrary convention; it arose from the intended use of PD to estimate “feature diversity” of sets of taxa (Faith, 1992a,Faith, 1992b). The 1992 companion paper (Faith, 1992b) expanded one of the examples from Faith, 1992a, in order to make clear the links from the PD definition to estimation of “feature diversity.” Here, I re-visit that example (Fig. 2) and highlight how it illustrated the link between PD’s feature diversity estimation and use of the root.

For sets of taxa, we can think of PD as capturing the new features arising along all those branches extending from the root of the tree and spanning all taxa. The example reproduced from Faith (1992b) illustrated one way to think about how those features are implicitly counted by PD (Fig. 2a,b). The tree (Fig. 2b) shows changes along the branches corresponding to the characters shown in (Fig. 2a). Here, we can think of these as changes from a 0 state in ancestral taxa, to some new 1 state for a number of characters, following the convention where ancestral or “outgroup” states/features are coded “0” and derived states coded “1” (Faith, 1992b). In explaining these worked PD examples, Faith (1992b) made it clear that “*the outgroup, “O” is also assumed to be protected.*” In this way, the example acknowledges the existence of the deeper history, and that outgroup taxa also “persist,” as a reference point for estimating feature diversity.

Using the marked tree, one can calculate the PD, and corresponding feature diversity score for any single taxon or subset of taxa from the tree. For example, taxon *j* would have a PD score of 4, indicating the 4 features/states arising along the branches to that taxon, so adding to the list of 0-state features contributed from the outgroup (Fig 2a,b). As indicated in the discussion above, this index value does not have to count all the features back to the root of all life. This index value for PD is based of the measure of branch lengths back to a root for this “complete” tree, and enables comparisons among different subsets of taxa.

These examples using two alternative features/states, and 0–1 codings, for characters are not the only way to describe the PD link to feature diversity. Nevertheless, these examples from Faith (1992b) highlight the role of the root in PD calculations of feature diversity. The estimated feature diversity of a set must take into account the fact that a root of the tree corresponds to ancestral or outgroup features. Put another way, only an unrealistic, hypothetical, case where a set of taxa are the only ones left on the planet would allow us to ignore the root or outgroup. At the extreme, if only one species was left on the planet, there would be little feature diversity. However, PD dismisses such a scenario as unrealistic and acknowledges that some outgroup taxon also exists, so that the diversification along the branches from that outgroup to the single species indicates feature diversity, as for taxon *j* above.

The final set of examples in Faith (1992b) further highlighted exactly this point. The examples (Fig. 4 in Faith, 1992b) focused on the use of classification/taxonomic information [the approach now taken-up in important web-based implementations of PD (http://www.deh.gov.au/biodiversity/abif/bat/technical.html)]. One example indicates how the PD of single species depends on reference to the root of the tree. Here, Faith (1992b) noted:

“interpreting the different taxonomic classes as separated (along phylogenetic paths) by nominated amounts of change means that the basic classification can be interpreted in the PD framework [Fig. 4(c)]: here, the contribution of species c is apparent because it is the only representative of a family-level taxon.”

These examples document the necessity to count the lengths of branches back to some common ancestor or root in order to properly credit the set of taxa as indicating relative feature diversity. While the definition of PD does not explicitly refer to the root of the tree, both the early examples and the stated rationale based on feature diversity highlight the link from the definition to the important consideration of some root of the tree.

### Reference to the correct PD definition in other early studies

We see that the early PD papers (Faith, 1992a,Faith, 1992b) not only provided the feature-diversity rationale for PD’s consideration of the root of the tree but also presented an illustrative example showing how PD, with its proper consideration of the root, is used comparatively. Crozier et al. (2006) therefore simply miss-represented the 1992a,b studies in claiming that “Faith’s original formulation (1992) clearly defined PD (“Phylogenetic diversity”) as including just the surviving species in a set, and did not include the root of the complete tree in this calculation.”

Crozier et al.’s statements in 2006 are all the more surprising given Crozier’s own published characterizations of the PD method some years’ earlier. For example, Crozier (1992) in referring to the Faith (1992a) study, noted that “Faith (this issue) proposes preserving that set of species which maximises the length of tree preserved.” It is hard to imagine this statement as implying anything other than the correct idea of PD of as set as capturing all the branches spanned by the set back to some base or root of the tree. This characterization by Crozier certainly does not seem to have been accidental. A few years’ later, Crozier et al. (1999) again cited Faith (1992a) and again said: “Phylogenetic diversity (PD) [7, 8] is the length of phylogenetic tree preserved.”

Other early references to the PD definition of Faith (1992a) similarly capture the key idea that PD extends to the base or root of the tree, in reflecting the full amount of evolutionary history “preserved”. For example, May (1994) referred to the Faith (1992a) paper and said:

“if we had some quantitative measure of the branch lengths within the phylogenetic tree of the group in question, we could unambiguously quantify the amount of IEH vested within a species by adding up the lengths of the branches which connect it *to the base of the tree and appropriately discounting all shared branches (Faith 1992*…”).

May (1994) went on to refer to this process, just as Crozier (1992) did, as “maximizing the summed branch length that was preserved.”

## II. The Definition of PD was not Changed by Faith and Co-workers

Crozier et al. (2006) argued that Moritz and Faith (1998) changed the definition of PD to include the distance to the root, following work by Nee and May (1997). The implication of this claim would be that Faith and Baker (2006) presented false information in stating that the original Faith (1992a) version of PD included the root. Given the description in section I, it will not be surprising to learn that the characterization by Crozier et al., not Faith and Baker, is incorrect. Here, as supporting evidence, I simply document the ongoing recitation by Faith and colleagues of the same definition, with continued reference back to Faith (1992a), over the past 15 years.

For example, Faith (1997) said: “A simple phylogenetic diversity measure, “PD,” estimates the relative feature diversity of a set of taxa by *the total length of the path spanned by these taxa* on the corresponding phylogenetic tree.”

Faith (2002) said: “representation of “evolutionary history” (Faith, 1994b) encompassing processes of cladogenesis and anagenesis is assumed to provide representation of the feature diversity of organisms. Specifically, the phylogenetic diversity (PD) measure estimates the relative feature diversity of any nominated set of species by *the sum of the lengths of all those phylogenetic branches spanned by the set* (Faith, 1992a, Faith, 1992b, Faith, 1994b).”

Faith et al. (2004) said: “the total PD of a given set is *the total phylogenetic branch length spanned* (represented) by its member species.”

Faith and Williams (2006) said: “A measure of phylogenetic diversity, “PD,” is defined as the minimum *total length of all the phylogenetic branches required to span a given set of taxa* on the tree (Fig. 1). Larger PD values imply greater expected feature diversity.”

Clearly, a survey of the literature demonstrates that Faith and colleagues have continued to put forward the same definition of PD.

In any case, it remains hard to understand why the study by Nee and May (1997) could be seen to have prompted any change in definition. Nee and May’s text made the typical reference to Faith (1992a) as providing an approach to address the amount of evolutionary history preserved:

“If k species out of a total of n are saved, it is natural to express the amount of history preserved as a fraction of the total amount that could have been preserved if all n species had been saved. How can this “amount of evolutionary history” be measured? For many purposes, it may be best simply to count species as such. But, as emphasized by Vane-Wright and others (1, 8), it is often useful to measure the loss at a more fundamental level…”

Here, Faith (1992a) was cited (their reference 8), and was linked to the idea of using phylogeny to quantify the amount of evolutionary history “preserved”. Far from somehow provoking a new definition, Nee and May (1997) simply echoed May’s earlier 1994 paper (see above) in referring to Faith (1992a).

I conclude that Faith and colleagues did not change the definition of PD, and continued to use the original 1992 definition in its proper sense as implying the inclusion of the root.

## Conclusion and Discussion

Faith and Baker (2006) correctly stated that Faith (1992a) put forward the original definition of PD as including the root. Crozier et al. (2006) incorrectly stated that Faith (1992a) defined PD as not using the root of the tree. Crozier et al. appear to have simply ignored the 1992 original examples showing how the root is considered, and how it links to the feature diversity rationale for PD.

Faith and Baker (2006) correctly stated that the definition has not been changed by Faith and colleagues over the past 15 years. Crozier et al. (2006) incorrectly stated that Faith and colleagues *changed* the definition in 1998. Crozier et al. appear simply to have ignored all the subsequent quotes of the same, original, definition, in the later papers over the past 15 years, by Faith and colleagues.

Ironically, when I presented the Faith (1992a,Faith 1992b) examples to colleagues shortly after publication, the criticism then was along the lines of “it’s not fair – PD only works because it uses the root.” The implication was that the restricted set viewed in isolation should somehow define the diversity measure for the set, with no need to look back at its evolutionary history. Clearly, the feature diversity rationale for PD shows why evolutionary history, and the root of the tree, do matter.

Those early discussions illustrate how it has been difficult to anticipate the possible confusions arising from the PD definition and to make timely clarifications. PD no doubt will continue to have a range of uses – and misuses. But at least the original 1992 papers provide nice examples that show how PD “works.” Hopefully, the further exposition of those examples presented here will help guide future PD applications. Surely, the risk in simply ignoring past work is that much effort might be expended in recreating methods, rather than exploring their exciting bio-informatics applications.
